# Idiopathic Pulmonary Fibrosis: Aberrant Recapitulation of Developmental Programs?

**DOI:** 10.1371/journal.pmed.0050062

**Published:** 2008-03-04

**Authors:** Moisés Selman, Annie Pardo, Naftali Kaminski

## Abstract

The authors discuss evidence suggesting that embryonic signaling pathways involved in epithelium/mesenchymal communication and epithelial cell plasticity may be aberrantly switched on in idiopathic pulmonary fibrosis.

Idiopathic pulmonary fibrosis (IPF) is a chronic, progressive, and usually lethal disease of uncertain etiology [1]. It has been proposed that IPF likely results from an aberrant activation of alveolar epithelial cells after injury that provoke the migration, proliferation, and activation of mesenchymal cells with the formation of fibroblastic/myofibroblastic foci, leading to the exaggerated accumulation of extracellular matrix with the irreversible destruction of the lung parenchyma [2–4]. The molecular mechanisms that determine the persistent nature of IPF are poorly understood. While aberrant activation of developmental pathways that are usually suppressed in adult tissues is often noticed in cancer, it is only rarely observed in non-malignant diseases. In the following pages we present evidence from gene expression studies and animal models of disease that suggest that IPF is characterized by aberrant activation of developmental pathways (Box 1).

**Box 1. Developmental Pathways Likely Involved in Idiopathic Pulmonary Fibrosis**

**The Wnt signaling pathway:** A signaling pathway that is involved in embryogenesis. Elimination of Wnt causes development of wingless fruit flies, hence the name Wnt (Wingless type). The central player in the signaling cascade of the canonical Wnt pathway is β-catenin. Nuclear β-catenin interacts with transcription factors such as LEF-1/T cell–specific transcription factor to affect transcription. Most Wnts work through specific receptors (Frizzled family of receptors and LRP5/6 co-receptors) and affect degradation and nuclear localization of β-catenin. In adults, dysregulation of the Wnt pathway leads to a variety of abnormalities and degenerative diseases, including cancer and fibrosis.

**TGF-β signaling:** The TGF-β superfamily of growth factors that includes TGF-β, activins, and BMPs regulates a plethora of biological processes during embryonic development as well as in adult life. A common mechanism through Smad activation applies to signaling mediated by all TGF-beta superfamily members. However, TGF-β ligands signal preferentially through Smad-2 and -3, whereas BMP signaling is mediated through Smad-1, -5, and -8. TGF-β and BMPs have opposite effects in a number of processes. Importantly, TGF-β has a profibrotic effect and induces epithelial–mesenchymal transition, while several BMPs display the contrary effect.

**PTEN/PI3 kinase/Akt signaling pathway:** The development and function of many tissues that feature tubular networks, including the lung, are regulated by intricate programs of epithelial cell morphogenesis. PTEN controls epithelial morphogenesis, integrating cellular polarity with tissue architecture. In the adult life, PTEN is an important tumor suppressor, and its dysfunction has been associated with cancer susceptibility. Importantly, PTEN becomes activated during wound healing, promoting fibroblast apoptosis. Decreased PTEN expression is found in IPF fibroblasts, which may enhance their survival, causing active fibroblastic foci persistence.

**Hedgehog signaling pathway:** A pathway critical in development. The pathway is named after its main ligand hedgehog. Sonic hedgehog is a morphogen that can specify multiple cell identities as a function of its concentration. In the lungs, Shh participates in branching morphogenesis, controlling bud size and shape. Lung-specific overexpression of Shh results in severe increase of interstitial tissue. Strong Shh expression has been identified in reactive alveolar epithelial cells in IPF lungs.

**Cross-talk pathways:** Emerging evidence supports the theory that the cell response to extrinsic signals relies not only on the effect of a single pathway, but on the integration of numerous signals from a plethora of cross-talking pathways. In this context, TGF-β/Smad, Wnt/β-catenin, BMP, PTEN, and members of the hedgehog and other families of secreted factors pathways operate through complex interconnections and feedback loops.

## Transcriptional Signatures of IPF Lungs Are Enriched with Developmental Genes

Microarray analysis identified an IPF-specific gene expression signature characterized by the up-regulation of genes indicative of an active tissue remodeling program, including extracellular matrix and a large number of myofibroblast/smooth muscle cell–associated and epithelial cell–related genes [3,5]. Recently, we have reanalyzed previously published datasets [3,5,6] using analytical approaches that allow global and unbiased mapping of the functional themes that characterize IPF lungs in comparison to controls or to other interstitial lung disease. The analyses revealed that IPF lungs were significantly enriched with genes associated with lung development [7]. The up-regulated development-relevant genes included several members of transcription factor families such as the Sry-related high mobility group box and forkhead box, and genes related to the Wnt/β-catenin pathway [6,7]. (For complete datasets see http://www.dom.pitt.edu/paccm/Genomics/data.htm).

While transcription factors active in morphogenesis and differentiation of the embryonic lung may be transiently expressed during adult lung repair, as in naphthalene injury [8], they are only rarely expressed in the normal lung. In fact, their sustained expression is often thought of as a marker for malignant transformation.

**Summary Points**

- Idiopathic pulmonary fibrosis is a devastating lung disorder of unknown etiology that inexorably leads to death in a relatively short time because of the lack of any effective therapy.
- IPF lungs are enriched with genes associated with lung development, indicating that embryonic signaling pathways involved in epithelium/mesenchymal communication and epithelial cell plasticity may be aberrantly activated in this disease.
- Developmental genes include members of transcription factor families such as the Sry-related high mobility group–box and forkhead box, and genes related to the Wnt/β-catenin pathway.
- Epithelial-to-mesenchymal transition, a key process in embryogenesis, may contribute to the fibroblast expansion in IPF lungs. Diverse molecular networks in IPF lungs seem to constitute an environment that drives epithelial cells to express mesenchymal cell properties.
- A better understanding of the dysfunctional activation of embryological pathways in IPF may result in novel, more effective therapeutic strategies.

## Epithelial Cell Plasticity

Epithelial cells are motile and can migrate away from their nearest neighbors [9]. However, under normal conditions they do not detach and move away from the epithelial layer. This arrangement can be disturbed by a process known as epithelial–mesenchymal transition (EMT). In the process of EMT, epithelial cells lose many of their epithelial characteristics and obtain properties that are distinctive of mesenchymal cells [10]. They become migratory, down-regulate the expression of cell adhesion molecules, primarily E-cadherin, lose their apical–basal polarity, and express mesenchymal molecules such as fibronectin and N-cadherin [11,12]. EMT is a key process in embryogenesis, where it leads to the formation of a migratory mesenchyme that progresses along the primitive streak and populates new areas of the embryo that will develop into mesoderm and endoderm [11].

## EMT May Contribute to the Fibroblast Expansion in IPF

An EMT-like process has been reported in cancer progression and metastasis, and in fibrotic disorders [13–23]. Recently, EMT was also observed in lung fibrosis by two groups that noticed numerous cells co-expressing epithelial and mesenchymal markers (thyroid transcription factor-1/α-smooth muscle actin [24] or surfactant protein C/N-cadherin [25]) within the expanded interstitium in IPF lungs. Moreover, using a triple transgenic mouse reporter system, Kim et al. demonstrated that EMT plays an important role during lung fibrogenesis and may be more widespread than previously thought [25].

While the mechanisms underlying EMT in IPF are still unclear, many EMT-related genes such as *transforming growth factor (TGF)*-β3 [26,27], *lymphoid enhancer factor-1 (LEF-1)* [28], and *Slug,* a TGF-β target gene required for EMT, as in the developing chicken heart [27], are up-regulated in IPF lungs (Table 1).

**Table 1:**
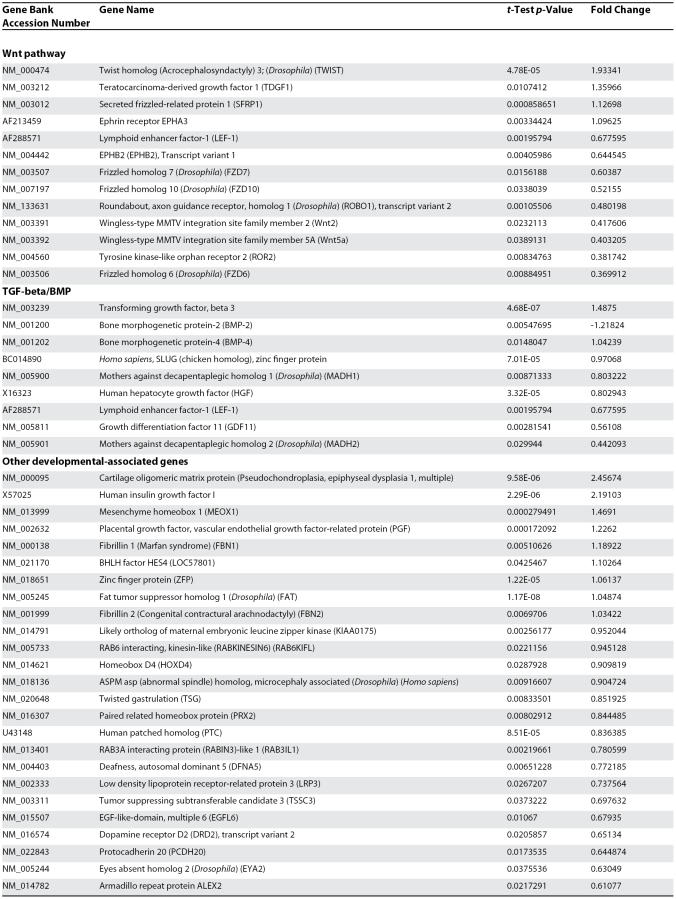
Developmental-Associated Differentially Expressed Genes in Idiopathic Pulmonary Fibrosis Compared With Normal Lungs

**Table 1:**
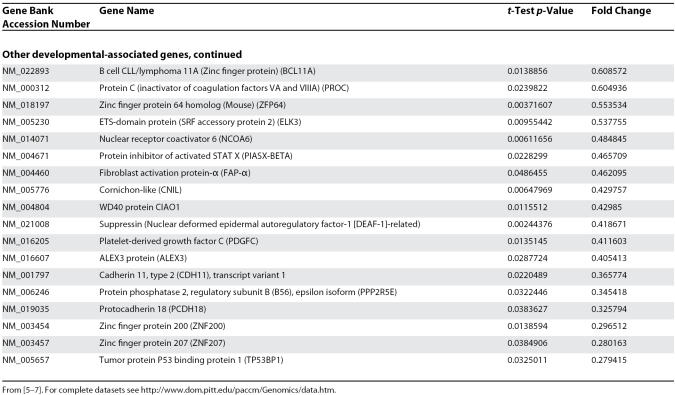
Continued.

## Bone Morphogenetic Proteins/TGF-β Balance and EMT

**Figure 1:**
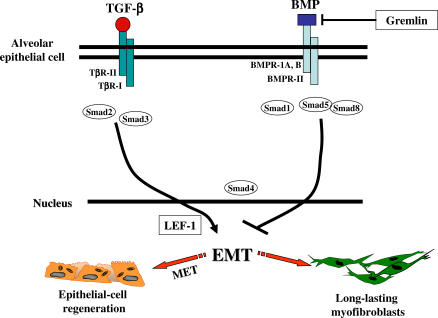
Schematic Overview of the Epithelial–Mesenchymal Transition as Modulated by TGF-β and BMPs In IPF lungs, TGF-β3, gremlin, and LEF-1 are up-regulated, while BMP-2 is down-regulated. Dysregulation of TGF-β/BMP pathway induces a fibrotic phenotype. EMT, so important in development, is likely to represent one possible response of the alveolar epithelial cell facing sustained injury. Increased expression of BMPs may not only antagonize TGF-β-induced EMT, enhancing re-epithelialization, but also may cause the opposite process, mesenchymal-to-epithelial transition (MET).

Bone morphogenetic proteins (BMPs) and TGF-β belong to a superfamily of multifunctional cytokines that includes different isoforms with highly specific functions including wound healing, extracellular matrix remodeling, and the control of epithelial– mesenchymal interactions during embryogenesis [29,30]. Importantly, BMPs antagonize the effects of TGF-β regarding EMT and induce the inverse process of mesenchymal-to-epithelial transition [10]. In tubular epithelial cells, BMP-7 reverses EMT by directly counteracting TGF-β-induced Smad-dependent cell signaling [31]. In kidney fibrosis, this antagonism may lead to regeneration of injured tissues, suggesting that reversal of EMT may have therapeutic advantages and that fibrosis may be reversible [31]. BMP-2 is decreased and BMP-4 is increased in IPF lungs, compared with controls (Table 1). More importantly, gremlin, the main BMP antagonist that modulates early limb outgrowth and patterning in the mouse embryo [32], is increased in IPF lungs [33]. Gremlin is also found in human diabetic nephropathy, where it colocalizes with TGF-β [34]. TGF-β induces gremlin expression in association with EMT in lung epithelial cells [33]. Taken together, these data suggest that increased TGF-β expression, decreased BMP-2 expression, and active BMP inhibition by gremlin create an EMT-favoring environment in IPF lungs (Figure 1).

The occurrence of EMT in the lung represents a dramatic shift in cellular phenotype and requires reversal of early embryonic programs. Unlike kidney development, where the tubular epithelium originates from cells that undergo mesenchymal-to-epithelial transition [35,36], in the lung the formation of the various cell types lining the proximal and distal airways occurs through the differentiation of the epithelial precursor cells [37]. In other words, it may be more “natural” for kidney epithelial cells to undergo EMT than for lung epithelial cells. In fact, *Snail* activation is sufficient to induce EMT and kidney fibrosis in adult transgenic mice [38]. Thus, EMT in IPF probably represents a dramatic reprogramming of epithelial cells [39].

## The Wnt Signaling Pathway

Wnts comprise a large family of secreted glycoproteins that activate multiple distinct types of intracellular signaling pathways through canonical and noncanonical Wnt pathways [40]. Wnt signaling regulates a wide range of developmental processes, and its aberrant activation can lead to disease [41,42]. Canonical Wnt signaling inhibits the phosphorylation and degradation of β-catenin, allowing its translocation into the nucleus and its interaction with the high mobility group domain–containing, DNA-binding proteins (including the previously mentioned LEF-1) to regulate target gene expression [40–42]. β-catenin influences epithelial cell differentiation in the lung, and is required for the normal differentiation of the bronchiolar and alveolar epithelium [43].

Several Wnt genes are expressed during lung development. Wnt7b-deficient mice exhibit impaired alveolar type I cell differentiation, have hypoplastic lungs, and die at birth of respiratory failure [44]. Similarly, Wnt5a-deficient mice also die shortly after birth from respiratory failure, but in contrast to Wnt7a-deficient mice, they exhibit increased proliferation of lung epithelial and mesenchymal compartments [45,46]. Interestingly, Wnt5a-null embryos showed increased expression of Sonic hedgehog (Shh), suggesting that Wnt5a signaling is required for the normal down-regulation of Shh, and that in the absence of Wnt5a, Shh-induced mesenchymal proliferation continues in late gestation. As will be discussed, epithelial expression of Shh has also been found in IPF lungs [47,48].

**Five Key Papers in the Field**

**Armanios et al., 2007** [59] Mutations of genes encoding telomerase reverse transcriptase and telomerase RNA support the idea that pathways leading to telomere shortening are involved in the pathogenesis of familial idiopathic pulmonary fibrosis.

**Selman et al., 2006** [3] This was the first paper to compare the gene expression profile of different interstitial lung diseases, providing evidence that idiopathic pulmonary fibrosis is characterized by a distinct transcriptional signature.

**Thiery and Sleeman, 2006** [10] In this detailed review on epithelial cell plasticity, the authors dissect the molecular events during which epithelial cells are transformed into mesenchymal cells and vice versa.

**Koli et al., 2006** [33] Findings suggest that overexpression of the BMP inhibitor gremlin may play a role in the pathogenesis of IPF, and may function to enhance the fibrotic response by modulating BMP-4 signaling in the lung.

**Willis et al., 2005** [24] The authors demonstrate for the first time the epithelial–mesenchymal transition in idiopathic pulmonary fibrosis.

## The Wnt Signaling Pathway in IPF

**Figure 2:**
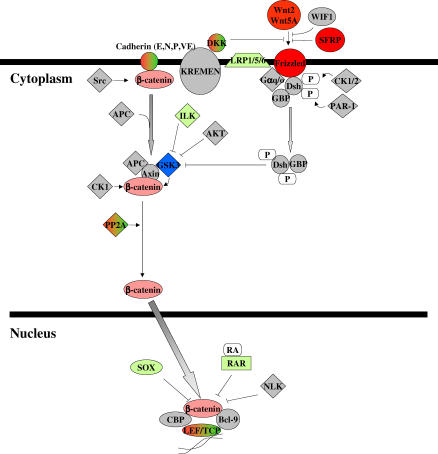
Changes in the Expression of Genes Known to be Involved in the Canonical Wnt Pathway The figure was generated using Ingenuity Pathways Analysis (Ingenuity Systems, http://www.ingenuity.com/). Increased genes are in increasing shades of red, decreased are in increasing shades of green. Combined red and green is when different members are changed in different directions.

Using gene expression microarrays, we demonstrated up-regulation of several members of the Wnt signaling pathway in IPF lungs, compared either with normal lungs or other interstitial lung diseases [3,5] (Table 1). For example, WISP-1 and the secreted frizzled-related protein 2 are increased in IPF compared with hypersensitivity pneumonitis [3]. Several other Wnt pathway-related genes are also overexpressed in IPF lungs compared to normal controls (Table 1; dataset used in [6]; Gene Expression Omnibus database serial accession number GSE2052). The overall balance of the Wnt pathway genes overexpressed in IPF lungs seems to favor activation of the canonic pathway (Figure 2). To date, there is only one study that directly demonstrated aberrant nuclear localization of β-catenin in bronchiolar/alveolar epithelial cells and in fibroblasts from the fibroblastic foci in IPF lungs [49]. Activation of β-catenin in epithelial cells is also indirectly corroborated by the overexpression of downstream genes such as *MMP-7* and *osteopontin* [5,6], and may also be related to EMT.

Taken together, these data suggest a significant role for the Wnt pathway in IPF.

## Fibroblast Expansion in IPF: Mirroring Aggressive Fibromatosis?

A key process in the development of IPF is the formation of the fibroblastic focus. It has been suggested that these foci represent discrete isolated foci of fibroblasts/myofibroblasts. However, using three-dimensional reconstruction of the IPF lungs, other studies have suggested that fibroblast foci are the leading edge of a complex reticulum that is highly interconnected extending from the pleura into the underlying parenchyma [50].

It is increasingly apparent that mesenchymal cells in fibroblastic foci in IPF exhibit a variety of abnormalities compared to normal lung fibroblasts or fibroblasts from other lung diseases. Some of these abnormalities are related to Wnt pathway signaling or to up-regulation of genes present in exaggerated fibroplasias. β-catenin stabilization (as observed in IPF) has been reported in hyperplastic cutaneous wounds, in keloid scars, and in aggressive fibromatosis, among other disorders [51–53]. Furthermore, transgenic mice that express stabilized β-catenin in mesenchymal cells develop aggressive fibromatosis suggesting a common role for β-catenin in abnormal fibrotic responses [52]. These findings raise the possibility that unchecked activation of a process important in normal wound healing causes abnormal fibroproliferative processes.

Aggressive fibromatosis is a soft tissue tumor composed of a clonal population of mesenchymal, spindle-shaped cells. It is locally invasive, but rarely metastasizes. Microarray analysis identified four genes uniquely overexpressed in this disorder: *ADAM12, WISP-1, SOX-11,* and *fibroblast activation protein-alpha (FAP*-a) [54]. WISP-1 and several members of the SOX family are up-regulated in IPF [3]. FAP-α is selectively expressed by a subset of fibroblasts in areas of ongoing tissue injury in IPF lungs, but not in normal lung tissue or in tissue with centriacinar emphysema [55]. FAP-α is a type II transmembrane serine protease expressed at sites of tissue remodeling in embryonic development [56,57]. FAP-α is highly expressed on reactive stromal fibroblasts in over 90% of human epithelial cancers, in healing wounds, and in sarcomas, but is not detected in fibroblasts of benign epithelial tumors or normal adult tissues [57,58].

An additional analogy between IPF and aggressive fibromatosis is the recent evidence that suggests involvement of pathways leading to telomere shortening in the pathogenesis of both disorders [59–61]. Telomeres are noncoding DNA sequences at the end of eukaryotic chromosomes that maintain chromosomal integrity and prevent replication of defective genes [62]. When normal cells reach a critical telomere length, they exit the cell cycle and undergo senescence. The putative relationship between telomerase and human disease is demonstrated in two opposite situations. On one side of the spectrum, most human cancers are characterized by the expression of telomerase, which helps to maintain telomere length and enhance indefinite cell proliferation [63]. On the other side of the spectrum, in pulmonary fibrosis and aggressive fibromatosis, telomerase activity seems to be diminished, with consequent premature telomere shortening. Two recent reports demonstrated that mutations in the genes encoding telomerase components are detected in patients with familial IPF, and occasionally in patients with sporadic IPF [59,60]. According to these studies, pulmonary fibrosis may be at least partially related to a telomerase deficiency and short dysfunctional telomeres, which after DNA damage leads to cell death or cell-cycle arrest. Interestingly, cigarette smoking, a strong environmental risk factor for IPF, causes telomere shortening in a dose-dependent manner [64]. Since alveolar epithelial cells play a critical role in the pathogenesis of IPF, and telomerase expression is generally restricted to cells with the capacity to proliferate, it has been speculated that fibrotic response in patients with short telomeres is provoked by a loss of alveolar cells [59], which may be enhanced by exposure to cigarette smoke.

Naturally, there are many differences between aggressive fibromatosis and the fibroblastic foci of IPF, primarily the clonality of cells; however, some of the pathways that are activated in the former may also participate in the latter.

## Phosphatase and Tensin Homologue Deleted on Chromosome 10

Phosphatase and tensin homologue deleted on chromosome 10 (PTEN) is a phosphatidylinositol phosphate phosphatase that is frequently deleted or inactivated in human cancers [65]. PTEN is critical in development, and PTEN-null embryos die early during embryogenesis [66,67].

Significant to our discussion, a critical role for PTEN in the orchestrated death and removal of myofibroblasts has been suggested. In response to collagen matrix contraction, PTEN triggers fibroblast apoptosis by leading to decreased PI3 kinase activity and Akt phosphorylation [68]. Myofibroblasts from IPF fibroblastic foci undergo decreased apoptosis compared to myofibroblasts from fibromyxoid lesions of cryptogenic organizing pneumonia, a potentially reversible lung disorder [69,70]. Impaired apoptosis may be at least partially associated with the decreased expression of PTEN observed in IPF lungs [71]. Additionally, since PTEN suppresses fibroblast differentiation to myofibroblasts [71], its reduced expression in IPF fibroblastic foci may have a dual deleterious effect, inducing myofibroblast differentiation and increasing survival.

Importantly, the PTEN/PI3 kinase/Akt signaling pathway is a critical regulator of the interconnection between the TGF-β/Smad, Wnt/β-catenin, and BMP pathways [72–75].

## Sonic Hedgehog

Sonic (Shh), Indian (Ihh), and Desert (Dhh) are the hedgehog family members in mammals. Shh is expressed in the developing lung epithelium, and its primary receptor, Patched-1 (Ptc), is found in mesenchymal cells. In vitro and in vivo studies have shown that Shh increases the proliferation of lung mesenchymal cells, up-regulating the expression of smooth muscle actin and myosin [45].

In developing mice, Shh produced by the epithelium stimulates mesenchymal cell proliferation and differentiation, and importantly, its overexpression causes an aberrant increase in the ratio of interstitial mesenchyme to epithelial tubules [76,77].

In this context, a pattern of strong Shh expression has been identified in reactive alveolar epithelial cells as well as in epithelial cells lining the honeycomb cysts in IPF lungs [47,48], while microarrays indicated up-regulation of its primary receptor (Table 1), suggesting that the Shh pathway is activated on in this disease.

## Therapeutic Implications

Identification and targeting of these abnormal mediators/pathways will eventually allow the development of therapeutic agents to control and hopefully achieve regression of IPF remodeling. Strategies designed to target the fibrogenic action of TGF-β with agents blocking its signaling pathway, such as the BMP-7, have led to repair of severely damaged renal tubular epithelial cells and to improvement of renal function and survival in mice with nephrotoxic serum nephritis [78]. Along the same line of thought, hepatocyte growth factor, which among other functions inhibits TGF-β-induced EMT, may also be a useful therapeutic approach. It was recently shown that in vivo hepatocyte growth factor gene transfer reduced bleomycin-induced pulmonary fibrosis, improving alveolar epithelial repair, and importantly, decreasing TGF-β1 expression [79].

**Glossary**

**Aggressive fibromatosis:** A disease characterized by the formation of tumors consisting of well-differentiated fibroblasts.

**Bone morphogenetic proteins:** A family of proteins that were originally described by their ability to cause bone and cartilage formation. Most of them belong to the TGF-β superfamily, and are implicated in various stages of embryonic development.

**Epithelial–mesenchymal transition:** The process in which an epithelial cell acquires characteristics often attributed to mesenchymal cells. It has been implicated in embryogenesis, cancer, and tissue fibrosis.

**Gene expression microarrays:** Methods that allow the parallel measurement of the expression of multiple transcripts even of complete genomes, usually by hybridization.

**Idiopathic pulmonary fibrosis:** A disease characterized by progressive scarring of the lungs, without any recognizable cause.

**Myofibroblast:** a fibroblast that has muscle cell–like properties such as contractility. These cells are important in wound healing and in fibrosis. In IPF, the cells form collections that are called myofibroblast foci.

**Telomerase:** An enzyme that adds DNA sequence repeats to the 3′ end of DNA in telomeric regions at the end of chromosomes. Telomerase mutations are associated with multiple inherited diseases, while abnormal telomerase activation has been implicated in cancer.

## Conclusion

Many pathways that play an essential role during embryological development are inactivated later in life, although some of them may be transiently expressed during adult repair. Aberrant activation of these pathways during adult homeostasis leads to pathological events resulting in cancer, but may also be associated with the development of idiopathic pulmonary fibrosis. Dysfunctional activation of embryological pathways regularly repressed in the adult life may explain the persistent nature of the disease. Although some progress into unraveling the pathogenic mechanisms involved in IPF has been made, many open questions remain, and virtually no effective treatment is currently available. Designing and implementing interventions that target these embryological pathways may be required to develop novel anti-IPF therapies and to significantly improve the outcome of IPF patients.

## Abbreviations

BMP: bone morphogenetic protein
EMT: epithelial–mesenchymal transition
FAP-α: fibroblast activation protein-α
IPF: idiopathic pulmonary fibrosis
LEF-1: lymphoid enhancer factor-1
PTEN: phosphatase and tensin homologue deleted on chromosome 10
Shh: Sonic hedgehog
TGF-β: transforming growth factor-β
